# 4-(4-Nitro­benz­yl)morpholine

**DOI:** 10.1107/S1600536811005964

**Published:** 2011-03-02

**Authors:** Ling-Ling Yang, Ren-Lin Zheng, Guo-Bo Li, Qi-Zheng Sun, Yong-Mei Xie

**Affiliations:** a Department of Applied Chemistry, College of Chemical Engineering, Sichuan University, Chengdu 610041, People’s Republic of China; b State Key Laboratory of Biotherapy and Cancer Center, West China Hospital, West China Medical School, Sichuan University, Chengdu 610041, People’s Republic of China; c West China School of Pharmacy, Sichuan University, Chengdu 610041, People’s Republic of China

## Abstract

In the title compound, C_11_H_14_N_2_O_3_, an inter­molecular inter­action between a nitro group O atom and a neighboring benzene ring helps to stabilize the crystal structure [N⋯centroid = 3.933 (2) Å]. No classical hydrogen bonds are observed in the crystal packing.

## Related literature

For the biological activity of 4-(4-nitro­benz­yl)morpholine derivatives, see: Lan *et al.* (2010 ▶); Bavetsias *et al.* (2010 ▶). For the synthesis, see: Tsou *et al.* (2008 ▶). For standard bond lengths, see: Allen *et al.* (1987 ▶).
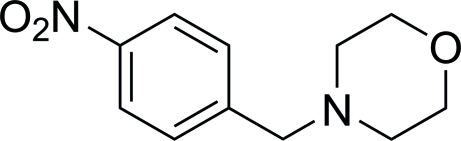


## Experimental

### 

#### Crystal data

C_11_H_14_N_2_O_3_
*M* *_r_* = 222.24
Monoclinic, 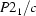

*a* = 6.1371 (2) Å
*b* = 8.2535 (4) Å
*c* = 21.9867 (9) Å
β = 94.929 (3)°
*V* = 1109.58 (8) Å^3^
*Z* = 4
Mo *K*α radiation
μ = 0.10 mm^−1^
*T* = 293 K
0.40 × 0.30 × 0.25 mm

#### Data collection

Oxford Diffraction Xcalibur Eos diffractometer
Absorption correction: multi-scan (*CrysAlis PRO*; Oxford Diffraction, 2006 ▶) *T*_min_ = 0.946, *T*_max_ = 1.0
6059 measured reflections
2264 independent reflections
1640 reflections with *I* > 2σ(*I*)
*R*_int_ = 0.018

#### Refinement

*R*[*F* ^2^ > 2σ(*F* ^2^)] = 0.044
*wR*(*F* ^2^) = 0.106
*S* = 1.04
2264 reflections
145 parameters
H-atom parameters constrained
Δρ_max_ = 0.12 e Å^−3^
Δρ_min_ = −0.13 e Å^−3^

### 

Data collection: *CrysAlis PRO* (Oxford Diffraction, 2006 ▶); cell refinement: *CrysAlis PRO*; data reduction: *CrysAlis PRO*; program(s) used to solve structure: *SHELXS97* (Sheldrick, 2008 ▶); program(s) used to refine structure: *SHELXL97* (Sheldrick, 2008 ▶); molecular graphics: *OLEX2* (Dolomanov *et al.*, 2009 ▶); software used to prepare material for publication: *OLEX2*.

## Supplementary Material

Crystal structure: contains datablocks I, global. DOI: 10.1107/S1600536811005964/vm2073sup1.cif

Structure factors: contains datablocks I. DOI: 10.1107/S1600536811005964/vm2073Isup2.hkl

Additional supplementary materials:  crystallographic information; 3D view; checkCIF report

## Figures and Tables

**Fig. 1.**
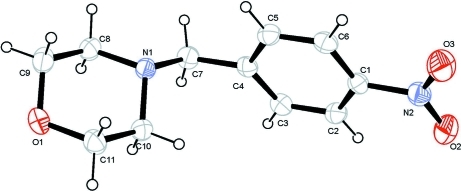
The molecular structure of the title compound with the atom-numbering scheme. Displacement ellipsoids are drawn at the 30% probability level.

**Fig. 2.**
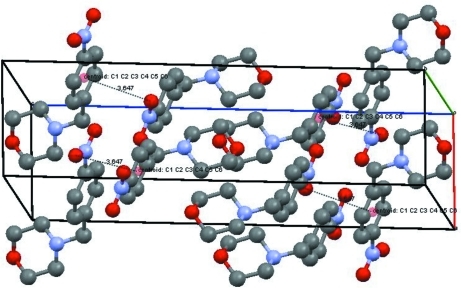
Packing diagram of the title compound showing the intermolecular interaction (dashed lines) between nitro group atom O3 and benzene ring C1-C6 (centroid indicated by pink sphere).

## supplementary crystallographic information

### Comment

4-(4-nitrobenzyl)morpholine derivatives are of great importance owing to their anticancer activity (Lan *et al.*, 2010; Bavetsias *et al.*, 2010). The title compound is one of the key intermediates in our synthetic investigations of anticancer drugs. In this paper, we synthesized the title compound and report its crystal structure. In the title compound, C_11_H_14_N_2_O_3_, (Fig. 1), the bond lengths and angles are within normal ranges (Allen *et al.*, 1987). The dihedral angle between the best planes between the phenyl and morpholino rings is 87.78 (10)°. An intermolecular interaction between the nitro group atom O3 and the benzene ring helps to stabilize the crystal structure. The distance O3···Cg(1)^a^ is 3.647 (2) Å (Fig.2, Cg(1) is the centroid of benzene ring C1-C6, symmetry operation (a): -*x*, -1/2+*y*, 1/2-*z*). There are no classical hydrogen bonds observed in the crystal packing.

### Experimental

The title compound was prepared by a method similar to that of Tsou *et al.* (2008). Crystals suitable for X-ray analysis were obtained by slow evaporation from a solution of dichloromethane.

### Refinement

All H atoms were positioned geometrically (C—H = 0.93–0.97 Å, N–H =0.91 Å) and allowed to ride on their parent atoms, with *U*_iso_(H) = 1.2*U*_eq_(parent).

### Crystal data

**Table:**

C_11_H_14_N_2_O_3_	*F*(000) = 472
*M**_r_* = 222.24	*D*_x_ = 1.330 Mg m^−^^3^
Monoclinic, *P*2_1_/*c*	Mo *K*α radiation, λ = 0.7107 Å
Hall symbol: -P 2ybc	Cell parameters from 2399 reflections
*a* = 6.1371 (2) Å	θ = 3.1–29.2°
*b* = 8.2535 (4) Å	µ = 0.10 mm^−^^1^
*c* = 21.9867 (9) Å	*T* = 293 K
β = 94.929 (3)°	Block, yellow
*V* = 1109.58 (8) Å^3^	0.40 × 0.30 × 0.25 mm
*Z* = 4	

### Data collection

**Table:**

Oxford Diffraction Xcalibur Eos diffractometer	2264 independent reflections
Radiation source: fine-focus sealed tube	1640 reflections with *I* > 2σ(*I*)
graphite	*R*_int_ = 0.018
Detector resolution: 16.0874 pixels mm^-1^	θ_max_ = 26.4°, θ_min_ = 3.1°
ω scans	*h* = −7→7
Absorption correction: multi-scan (*CrysAlis PRO*; Oxford Diffraction, 2006)	*k* = −8→10
*T*_min_ = 0.946, *T*_max_ = 1.0	*l* = −25→27
6059 measured reflections	

### Refinement

**Table:**

Refinement on *F*^2^	Primary atom site location: structure-invariant direct methods
Least-squares matrix: full	Secondary atom site location: difference Fourier map
*R*[*F*^2^ > 2σ(*F*^2^)] = 0.044	Hydrogen site location: inferred from neighbouring sites
*wR*(*F*^2^) = 0.106	H-atom parameters constrained
*S* = 1.04	*w* = 1/[σ^2^(*F*_o_^2^) + (0.0423*P*)^2^ + 0.1325*P*] where *P* = (*F*_o_^2^ + 2*F*_c_^2^)/3
2264 reflections	(Δ/σ)_max_ < 0.001
145 parameters	Δρ_max_ = 0.12 e Å^−^^3^
0 restraints	Δρ_min_ = −0.13 e Å^−^^3^

### Special details

**Table:**

Geometry. All esds (except the esd in the dihedral angle between two l.s. planes) are estimated using the full covariance matrix. The cell esds are taken into account individually in the estimation of esds in distances, angles and torsion angles; correlations between esds in cell parameters are only used when they are defined by crystal symmetry. An approximate (isotropic) treatment of cell esds is used for estimating esds involving l.s. planes.
Refinement. Refinement of F^2^ against ALL reflections. The weighted R-factor wR and goodness of fit S are based on F^2^, conventional R-factors R are based on F, with F set to zero for negative F^2^. The threshold expression of F^2^ > 2sigma(F^2^) is used only for calculating R-factors(gt) etc. and is not relevant to the choice of reflections for refinement. R-factors based on F^2^ are statistically about twice as large as those based on F, and R- factors based on ALL data will be even larger.

### Fractional atomic coordinates and isotropic or equivalent isotropic displacement parameters (Å^2^)

**Table:**

	*x*	*y*	*z*	*U*_iso_*/*U*_eq_	
O1	0.40024 (19)	0.31753 (14)	0.53296 (5)	0.0556 (3)	
O2	−0.4376 (2)	−0.42771 (17)	0.28701 (7)	0.0751 (4)	
O3	−0.1642 (2)	−0.57082 (16)	0.32340 (8)	0.0808 (5)	
N1	0.33645 (19)	0.14840 (16)	0.41946 (6)	0.0417 (3)	
N2	−0.2491 (2)	−0.44089 (19)	0.30912 (6)	0.0530 (4)	
C1	−0.1173 (2)	−0.29260 (19)	0.31894 (7)	0.0413 (4)	
C2	−0.2096 (2)	−0.1454 (2)	0.30324 (7)	0.0461 (4)	
H2	−0.3521	−0.1392	0.2853	0.055*	
C3	−0.0870 (2)	−0.0064 (2)	0.31460 (7)	0.0467 (4)	
H3	−0.1484	0.0941	0.3044	0.056*	
C4	0.1268 (2)	−0.0148 (2)	0.34109 (7)	0.0420 (4)	
C5	0.2166 (2)	−0.1668 (2)	0.35492 (7)	0.0468 (4)	
H5	0.3605	−0.1743	0.3718	0.056*	
C6	0.0965 (3)	−0.3056 (2)	0.34414 (7)	0.0471 (4)	
H6	0.1576	−0.4066	0.3536	0.057*	
C7	0.2629 (3)	0.1353 (2)	0.35452 (7)	0.0493 (4)	
H7A	0.3893	0.1325	0.3309	0.059*	
H7B	0.1771	0.2302	0.3421	0.059*	
C8	0.5018 (2)	0.2751 (2)	0.43006 (8)	0.0499 (4)	
H8B	0.4409	0.3785	0.4162	0.060*	
H8A	0.6256	0.2513	0.4069	0.060*	
C9	0.5767 (3)	0.2851 (2)	0.49693 (9)	0.0550 (5)	
H9A	0.6451	0.1835	0.5100	0.066*	
H9B	0.6853	0.3701	0.5033	0.066*	
C10	0.1559 (2)	0.1839 (2)	0.45620 (8)	0.0502 (4)	
H10A	0.0466	0.0991	0.4507	0.060*	
H10B	0.0879	0.2855	0.4430	0.060*	
C11	0.2381 (3)	0.1952 (2)	0.52265 (8)	0.0566 (5)	
H11B	0.1165	0.2191	0.5466	0.068*	
H11A	0.2992	0.0916	0.5361	0.068*	

### Atomic displacement parameters (Å^2^)

**Table:**

	*U*^11^	*U*^22^	*U*^33^	*U*^12^	*U*^13^	*U*^23^
O1	0.0660 (7)	0.0481 (7)	0.0523 (7)	−0.0032 (6)	0.0017 (6)	−0.0124 (6)
O2	0.0609 (8)	0.0800 (10)	0.0819 (10)	−0.0136 (7)	−0.0089 (7)	−0.0130 (8)
O3	0.0861 (10)	0.0489 (9)	0.1079 (12)	−0.0023 (7)	0.0107 (8)	0.0089 (8)
N1	0.0422 (7)	0.0462 (8)	0.0372 (7)	−0.0052 (6)	0.0053 (5)	−0.0017 (6)
N2	0.0584 (9)	0.0565 (10)	0.0451 (8)	−0.0045 (7)	0.0101 (7)	−0.0050 (7)
C1	0.0460 (9)	0.0465 (10)	0.0314 (8)	0.0000 (7)	0.0045 (6)	−0.0033 (7)
C2	0.0416 (8)	0.0573 (11)	0.0385 (9)	0.0041 (7)	−0.0026 (7)	−0.0006 (8)
C3	0.0516 (9)	0.0469 (10)	0.0408 (9)	0.0081 (7)	−0.0002 (7)	0.0019 (7)
C4	0.0474 (9)	0.0495 (10)	0.0294 (8)	0.0016 (7)	0.0055 (6)	−0.0017 (7)
C5	0.0399 (8)	0.0607 (12)	0.0394 (9)	0.0034 (8)	−0.0001 (7)	−0.0014 (8)
C6	0.0502 (9)	0.0476 (10)	0.0435 (9)	0.0104 (7)	0.0044 (7)	0.0016 (8)
C7	0.0542 (9)	0.0555 (11)	0.0386 (9)	−0.0047 (8)	0.0065 (7)	0.0027 (8)
C8	0.0463 (9)	0.0492 (11)	0.0546 (11)	−0.0072 (7)	0.0059 (7)	0.0007 (8)
C9	0.0523 (10)	0.0501 (11)	0.0610 (12)	−0.0059 (8)	−0.0047 (8)	−0.0050 (9)
C10	0.0457 (9)	0.0588 (11)	0.0468 (10)	−0.0069 (7)	0.0080 (7)	−0.0067 (8)
C11	0.0632 (11)	0.0609 (12)	0.0472 (10)	−0.0092 (9)	0.0128 (8)	−0.0105 (9)

### Geometric parameters (Å, °)

**Table:**

O1—C9	1.421 (2)	C5—H5	0.9300
O1—C11	1.4218 (19)	C5—C6	1.372 (2)
O2—N2	1.2210 (17)	C6—H6	0.9300
O3—N2	1.2214 (17)	C7—H7A	0.9700
N1—C7	1.4641 (19)	C7—H7B	0.9700
N1—C8	1.4616 (19)	C8—H8B	0.9700
N1—C10	1.456 (2)	C8—H8A	0.9700
N2—C1	1.473 (2)	C8—C9	1.504 (2)
C1—C2	1.372 (2)	C9—H9A	0.9700
C1—C6	1.384 (2)	C9—H9B	0.9700
C2—H2	0.9300	C10—H10A	0.9700
C2—C3	1.383 (2)	C10—H10B	0.9700
C3—H3	0.9300	C10—C11	1.507 (2)
C3—C4	1.3911 (19)	C11—H11B	0.9700
C4—C5	1.393 (2)	C11—H11A	0.9700
C4—C7	1.509 (2)		
			
O1—C9—C8	111.83 (13)	C4—C3—H3	119.6
O1—C9—H9A	109.3	C4—C5—H5	119.4
O1—C9—H9B	109.3	C4—C7—H7A	109.3
O1—C11—C10	111.71 (14)	C4—C7—H7B	109.3
O1—C11—H11B	109.3	C5—C4—C7	119.67 (13)
O1—C11—H11A	109.3	C5—C6—C1	118.75 (15)
O2—N2—O3	123.32 (15)	C5—C6—H6	120.6
O2—N2—C1	118.31 (15)	C6—C1—N2	118.95 (15)
O3—N2—C1	118.38 (14)	C6—C5—C4	121.20 (14)
N1—C7—C4	111.73 (13)	C6—C5—H5	119.4
N1—C7—H7A	109.3	H7A—C7—H7B	107.9
N1—C7—H7B	109.3	C8—N1—C7	111.09 (12)
N1—C8—H8B	109.6	C8—C9—H9A	109.3
N1—C8—H8A	109.6	C8—C9—H9B	109.3
N1—C8—C9	110.15 (14)	H8B—C8—H8A	108.1
N1—C10—H10A	109.6	C9—O1—C11	109.49 (13)
N1—C10—H10B	109.6	C9—C8—H8B	109.6
N1—C10—C11	110.07 (13)	C9—C8—H8A	109.6
C1—C2—H2	120.6	H9A—C9—H9B	107.9
C1—C2—C3	118.87 (14)	C10—N1—C7	111.73 (12)
C1—C6—H6	120.6	C10—N1—C8	108.57 (13)
C2—C1—N2	119.30 (14)	C10—C11—H11B	109.3
C2—C1—C6	121.76 (15)	C10—C11—H11A	109.3
C2—C3—H3	119.6	H10A—C10—H10B	108.2
C2—C3—C4	120.88 (15)	C11—C10—H10A	109.6
C3—C2—H2	120.6	C11—C10—H10B	109.6
C3—C4—C5	118.51 (15)	H11B—C11—H11A	107.9
C3—C4—C7	121.81 (15)		
